# Maintenance issues of elderly patients requiring nursing care with implant treatments in dental visiting: position paper

**DOI:** 10.1186/s40729-022-00458-8

**Published:** 2022-12-08

**Authors:** Chikahiro Ohkubo, Noriharu Ikumi, Yuji Sato, Mai Shirai, Kazuhiro Umehara, Isao Ohashi, Hirokazu Shibagaki, Yoshimine Niki, Chihiro Masaki, Itaru Mikami, Hiroshi Murakami, Osamu Yoshinaga, Masahiro Wada, Fumihiko Watanabe

**Affiliations:** 1 Department of Removable Prosthodontics, Tsurumi University School of Dental Medicine, 2-1-3 Tsurumi, Tsurumi-Ku, Yokohama, Kanagawa 230-8501 Japan; 2 Kanto-Koshinetu Brunch (Clinical Implant Society of Japan), 457-3 Iizuka-machi, Takasaki-shi, Gunma-ken, 370-0069 Japan; 3 Department of Geriatric Dentistry, Showa University School of Dentistry, 1-5-8, Hatanodai Shinagawa-Ku, Tokyo, 142-8555 Japan; 4 Touhoku-Hokkaido Brunch (Aomori Implant Research Group), 123, Dotemachi, Hirosaki, Aomori 036-8182 Japan; 5 Kanto-Koshinetu Brunch (Japan Institute for Advanced Dentistry), 4F Shiba TK Building, 1-8-25 Shiba, Minato-Ku, Tokyo, 105-0014 Japan; 6 Chugoku-Shikoku Brunch (Clinical Implant Society of Japan), 1-43-9, 4F Komagome TS Building, Komagome, Toyoshima-Ku, Tokyo, 170-0003 Japan; 7 Division of Oral Reconstruction and Rehabilitation, Kyushu Dental University, 2-6-1, Manazuru, Kokura-Kita, Kitakyusyu, Fukuoka 803-8580 Japan; 8 Touhoku-Hokkaido Brunch (Institute for Hokkaido Plastic Dentistry), 2F ARCS, 9-1-1, Kita24jonishi, Kita-Ku, Sapporo, Hokkaido 001-0024 Japan; 9 Department of Fixed Prosthodontics and Oral Implantology, School of Dentistry, Aichi Gakuin University, 2-11 Suemoridori, Chikusa-ku, Nagoya, Aichi 464-8651 Japan; 10 Kyushu Branch, Matsubasemachiurakawachi, Uki, Kumamoto, 869-0521 Japan; 11 Department of Prosthodontics and Oral Rehabilitation, Osaka University Graduate School of Dentistry, 1-8, Yamada-Oka, Suita, Osaka 565-0871 Japan; 12 Department of Crown and Bridge Prosthodontics, The Nippon Dental University School of Life Dentistry at Niigata, 1-8, Hamauracho, Chuo-Ku, Niigata, Niigata 951-8580 Japan

**Keywords:** Dental implant, Dental visiting, Elderly people needing long-term care

## Abstract

**Purpose:**

Japan, with an increasing number of elderly people needing long-term care in a super-aged society, urgent needs to develop the clinical guidelines on implant maintenance for elderly people with declining independence. The purpose is to categorize the troubles encountered in the care of patients with dental implants and to indicate actual practices and points of note.

**Methods:**

From the members of the Japanese Society of Oral Implantology, 12 expert panelists who were experienced with many problems of implant maintenance during dental visits and were familiar with their solutions were selected. Through repeated discussions in the many panel meetings, the problems of implant maintenance during dental visits were distilled.

**Results:**

During a dental visit, the oral cavity, general conditions, and background of elderly patients who cannot orally care for themselves must be grasped, and medical staff, care managers, and patients should understand the changes in these factors as time goes by. The solutions and responses that can be made differ greatly depending on the medical care facilities, the environment, differences in the experience of medical staff, and the patient’s background. Thus, it is necessary to select safe treatments appropriate to each situation.

**Conclusions:**

This paper features many opinions based on clinical experiences. However, clinical guidelines on implant management during dental visits should be formulated in the future based on the accumulation of evidence through the implementation of clinical research.

## Background

In Japan, which has developed into a super-aging society, the number of elderly people who require nursing care is increasing. For many of these people, visits to dentists and dental health management by themselves become more difficult than for others. The number of patients with dementia, which is a considerable risk factor for these elderly people, is estimated to exceed 7 million in Japan by 2025, accounting for 1 in 5 people aged 65 and older, according to the Ministry of Health, Labour and Welfare. Accordingly, the number of elderly people who cannot manage their own oral care will inevitably increase, while patients who currently benefit from improved QOL by implant treatments will become elderly and will themselves require nursing care in the near future. That is, implants which helped patients to eat while they were young and healthy may pose a high risk when they become elderly and require nursing care as lesions develop around the implants, or they may bite soft tissues in the mouth by mistake, and so forth. A further problem is that patients’ family members and the staff of care facilities do not have sufficient accurate knowledge of caring for implant prostheses.

There are few reports providing evidence of implant troubles in elderly people who require nursing care: no more than 15 reports can be found in a search of PubMed or the Igaku Chuo Zasshi (ICHUSHI) database as of January 2018 [1, 2]. Accordingly, we decided to form a consensus based on clinical experience by selecting an expert panel of clinicians with much experience of implant treatment in visiting dental care and conducting panel discussions.

## Methods

We decided to form a consensus based on clinical experience by selecting an expert panel of clinicians with much experience of implant treatment in visiting dental care and conducting panel discussions to prepare a position paper. We collected information from 1254 specialists and representatives of the Japanese Society of Oral Implantology. In beginning of 2017, we aimed for approximately equal numbers of clinicians from universities and practitioners, as well as by region. This expert panel of 12 members was commissioned to prepare the draft for this paper.

In August 2017, we held a panel meeting with the entire expert panel present to discuss the problems in implant treatment in visiting dental care, countermeasures against them, and reached a general consensus.

The draft of the paper was allocated to and written by all members of the expert panel, and a final consensus was obtained through many revisions and discussions by e-mail among the panel members. The final draft was completed after peer review by the Research Promotion Committee and the council of the Japanese Society of Oral Implantology in end of 2018. For the literature search, we used PubMed and the online database of Igaku Chuo Zasshi (ICHUSHI) as well as a manual search. There was no systematic review.

### Current state of visiting dental treatments

**Fig. 1:**
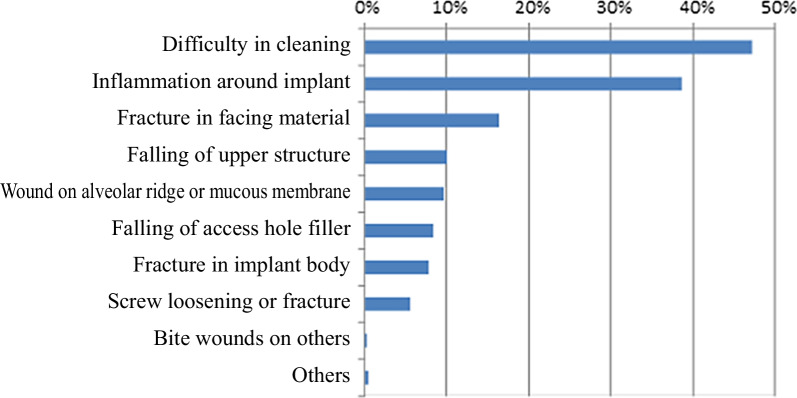
Types of implant-related troubles in visiting dental care

**Fig. 2:**
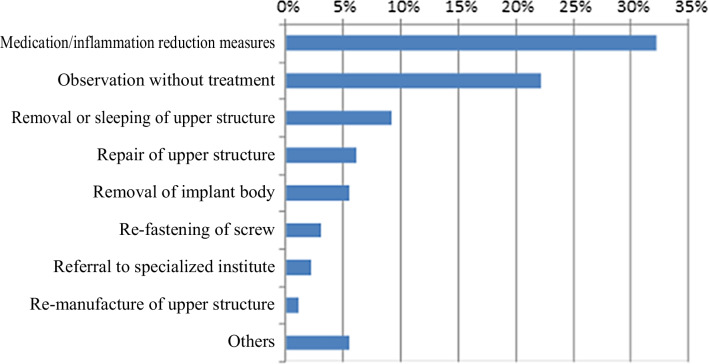
Measures against implant-related troubles in visiting dental care

1. Current and actual state of aging in Japan and the world
  The rate of aging in Japan has increased since around 2000 due to the aging population combined with the declining birthrate, and the country now has one of the highest aging levels in the world [3]. The stages of aging are referred to by the terms “aging society” (rate of aging 7% or higher), “aged society” (14% or higher), and “super-aging society” (21% or higher), and Japan is currently considered a super-aging society. In the Japanese Society of Oral Implantology, 29% of dentists (members) provided visiting dental care [4].
2. Current state of implants in elderly people and people who require nursing care
  In overseas countries, the rate of implant patients aged 70 or higher increased dramatically from 2002 to 2014 (from 7.7 to 21.0%) [1, 5, 6]. According to surveys of dental disease in fiscal 2011 and 2016 [7, 8], the number of elderly patients with implants increased from fiscal 2011 to 2016.
  In visiting dental care, 360 patients (2.9%) out of 12,356 admitted patients or patients at home had implants [4], of whom one-third had been given implants by the dentist who also provided the visiting dental care. In addition, more than half of these patients were incapable of self-care. The most common troubles involving implants were 47% with cleaning difficulties and 39% with inflammation of surrounding tissues (Fig. 1), and they were mostly treated with medication (32%) or kept under observation (22%) (Fig. 2).

### Systemic and local evaluation

1. **Nursing level and necessity of examination**
  Normally, systemic and mental frailty develops with age, making it difficult to walk and increasing the risk for onset of dementia. In addition, elderly people requiring a high level of nursing care end up being unable to perform oral care by themselves due to physical malfunctions or dementia. That is, oral hygiene worsens as the level of nursing care increases, and the patients require oral hygiene management.
2. **Diagnosis and inspection**
  1. Oral hygiene conditions
    The state of oral hygiene in the entire oral cavity should be examined to make a rough evaluation of the oral cleaning conditions. While the objective of oral hygiene management must be appropriate for the nursing level and instructions on cleaning to achieve the objective will be required, it is necessary to give proper information to those who provide nursing care in consideration of the living environment of the patient.
  2. Examination of tissues surrounding the implant
    Since there are more cases of dementia and physical restrictions as the nursing level increases, the health of the mucosal tissue surrounding the implant should be grasped and how oral hygiene may worsen with the progression of disease in the future should be predicted while the nursing level is still low and it is easier to obtain the patient’s understanding. While examination in accordance with periodontal treatment is necessary, there are many restrictions in visiting dental care including the portability of inspection devices, understanding of the patient, and difficulty in examination due to involuntary movements. Therefore, the disease state and rate of progression must be grasped and diagnosed based on minimum examinations.
    However, this information is often lacking for patients who received implant treatment at a different clinic or hospital, which may result in difficulties during maintenance.
  3. State of prostheses
    When examining a prosthesis during visiting dental care, the implant position, the contour of the prosthesis, the mode of connection between the implant body and prosthesis and so forth should be determined. If it is a fixed prosthesis, the occlusion and contact conditions should be inspected in addition to the mobility of the superstructure and clogging of the access hole. For implant overdentures, occlusion, fitting, fracture of denture, condition of wear of artificial teeth, fracture of attachment and so forth should be inspected in a similar manner to examination of normal dentures.
3. **X-ray examination**
  X-ray examination is important in diagnosing implants, and a portable X-ray device can dramatically improve diagnostic accuracy. If it is impossible to use X-ray examination at the location of care, it may be necessary to transport the patient to a medical facility to conduct an X-ray examination.
4. **Importance of coordination with other parties and information**
  To provide care for elderly people who require nursing care, coordination among many different specialists related to home care is important.

### Measures against various troubles

**Fig. 3:**
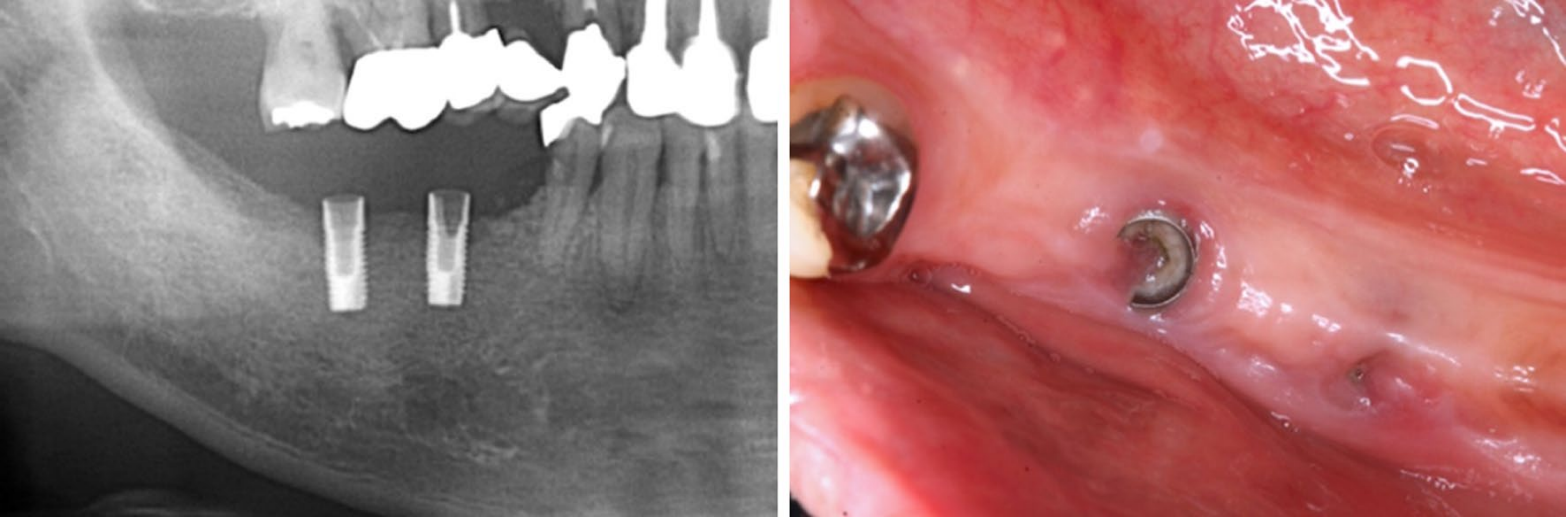
Fracture in neck part of implant

Since patients receiving visiting dental care are diverse, and often have systemic or mental problems, measures should take into consideration the following points if there are implant troubles (Table 1):

1. **Inflammations around implants in visiting dental care**
  1. Characteristics of inflammations around implants
    1. Acute peri-implantitis: reddening, swelling, bleeding, or drainage in soft tissues surrounding the implant, and strong pain caused by acute inflammation.
    2. Chronic peri-implantitis: chronic loss of bone around the implant, and persistent drainage and bleeding.
  2. Diagnostic methods for peri-implantitis
    While the examination items for peri-implantitis include the bacteriological test, plaque index (PI), probing pocket depth (PPD), bleeding on probing (BOP), implant mobility and X-ray examination [9].
    1. Visual examination
    2. Inflammation is diagnosed by pressing on and abrading the mucous membrane around the implant.
  3. Methods for handling peri-implantitis
    1. Reinforcement of brushing: explanation on the conditions inside the oral cavity (including implants) to the family members or care providers and instruction on oral hygiene methods to improve the oral cavity cleaning [10, 11].
    2. Mechanical cleaning (CIST: A): removal of plaque, food residue and dental calculus from around the implant.
    3. Sterilization treatment (CIST: B): local cleaning of the area around the implant using antibacterial agents.
    4. Antibacterial agent treatment (CIST: C): systemic administration of antibacterial agents or local administration of antibacterial agents with sustained release properties.
    5. When deep spreading of peri-implantitis does not abate: measures by visiting care become impossible, requiring treatment at a clinic including surgical treatment, removal or sleeping of the implant, and modification of the implant prosthesis.
2. **Implant fracture**
  1. Fracture in neck part of implant
    Fracture in the neck part of an implant mostly occurs due to mobility of the superstructure (also including abutment) caused by loosening, and separation of the superstructure occurs as a result (Fig. 3). Measures vary depending on whether the superstructure can be reattached when the range of fracture is small, whether a healing abutment is available for the type of implant is when it is clear, and so forth. If reattachment of the superstructure or attachment of a healing abutment is possible, a follow-up examination should be performed, carefully checking for mucositis caused by the sharp edges of the fractured implant. If it is difficult to seal the connection parts, they should be sealed with a self-curing resin, composite resin and so forth.
  2. Fracture in stem part of implant
    Mobility including the superstructure is observed when fracture occurs in the stem part. If X-ray images are not available, it must be determined whether the mobility is caused by loosening of the abutment screw, fracture in the stem part of the implant, or loss of integration.
    If a fracture has occurred in the implant stem or integration is lost, it can be removed easily by pulling. As it is difficult to remove the implant fractures remaining in the bone, the patient should be subjected to follow-up examination in this state.
3. **Screw loosening or fracture**
  1. Screw loosening
    Screw loosening appears clinically as mobility in the superstructure. Therefore, differentiating diagnosis based on whether only the superstructure is mobile or the implant itself is mobile is important as described in “Systemic and local evaluation” section. It is assumed that there is loosening in abutment screw if the fulcrum of mobility is palpated immediately below the edge of the mucous membrane, but the measures vary depending on whether the cement fixation method or screw fixation method is used. In either case, rotary cutting devices (engines) and a multi-driver set are usually necessary.
  2. Screw fracture
    Screw fracture mostly occurs when the superstructure has already been separated. There are various possible cases, including the case where a fractured screw is visible, and the case where it is not visible at all as fracture occurs deep inside the implant. If the fractured screw cannot be removed, it should be sealed with a self-curing resin, composite resin, stopping and so forth before the patient undergoes follow-up examination.
4. **Fracture in facing part**
  The normal facing repair processes are often difficult to implement in visiting dental care.
  1. Anterior teeth
    Since there are appearance issues and operations are relatively easy for anterior teeth, normal metal primer treatment, composite resin repair, as well as recontouring and polishing are possible.
  2. Molar teeth
    The causes of fracture in molar teeth include inappropriate occlusal relationship, occlusal interference and so forth caused by long-term changes in oral cavity conditions. Fracture often recurs if it is repaired temporarily without resolving and improving the fundamental cause. Therefore, treatment should involve only grinding the sharp edges of the fractured part using a rotary cutting device with follow-up examination instead of repairing it by force.
5. **Loss of temporary sealing on access hole**
  1. Cause and method of diagnosis
    Loss of temporary sealing in the superstructure with the screw fixation method occurs due to deterioration of the sealing material, occlusal wear, quenching or bruxism [12]. Stopping, photopolymerizing temporary sealing material, photopolymerizing ionomer filler, or photopolymerizing resin filler are used as temporary sealing materials, and inspection for mobility or subsidence in the temporary sealing material should be performed using a probe, followed by an attempt to implement removal and temporary sealing again.
  2. Measures: selection of temporary sealing material
    Materials that are soft and easily worn by occlusion such as stopping should be avoided. Even when providing a temporary sealing of multiple layers using swabs and temporary sealing materials, the temporary sealing material on the outermost layer should be a hard material which is resistant to deterioration, such as a hard resin. If the patient has a tendency for occlusal wear or clenching, the patient should be asked to use a night guard to avoid direct loads on the superstructure [13].
6. **Lack of retention of the prosthesis**
  1. Causes and method of diagnosis
    Falling of the superstructure is often observed in implants which adopt the cement fixation method. Common causes include using an abutment whose tooth crown vertical dimension is too small to ensure sufficient retentive force, deterioration of the cement for temporary cementation, and continuous application of abnormal occlusal force [14]. Normally, it is important to inspect for mobility or lift in the superstructure during regular check-ups.
  2. Measures: occlusal adjustment and cementation or change in superstructure
    Before permanently fixing the superstructure with the cement fixation method, the occlusal state, occlusal wear and whether there are habits such as clenching should be checked to consider whether to use a night guard and to provide instructions on mastication. If it is considered that cement fixation will not deliver sufficient retentive force as the intermaxillary space is too small, the superstructure should be changed to use the screw fixation method [12, 14–16].
7. **Poor cleaning**
  1. Causes and methods of inspection
    Possible causes of poor cleaning include inappropriate contour of the superstructure, shape of the interdental part, defective pontic shape, mucous membrane under the pontic, and bone resorption.
    Normally, the parts where poor cleaning is occurring should be checked using a disclosing agent. When the superstructure is manufactured after the patient reaches an elderly age, it is better to correct the shape to one that facilitates cleaning rather than focusing on appearance.
  2. Measures: professional care and instructions on cleaning
    It is important to give instructions on using auxiliary cleaning instruments (interdental brush, etc.) to nursing care providers to improve their cleaning skill. If the condition still does not improve, professional care by a dental hygienist should be provided along with proper cleaning instructions to the care providers.
8. **Wounds (bite wounds on lips, buccal mucosa, and paired mucous membrane)**
  1. Causes
    The superstructure itself can act as a weapon on the surrounding soft tissue including the lips and cheeks when the superstructure of an implant is left [17]. In addition, damages to the surrounding soft tissue may be caused by the implant body or abutment if it is left unattended after the superstructure falls off due to screw fracture and so forth, as described in “Limitations” section.
  2. Measures: removal of the cause
    The superstructure should be removed if it can be done easily, and the shape of the sharp edges which can cause wounds should be corrected if removal is difficult. If the condition still does not improve, it is necessary to consider cutting the superstructure, and if the systemic disorder of the patient permits, to consider removal or sleeping of the implant body at a specialized institute.
9. **Bite wounds on others**
  1. Causes
    Although rare (1%), some people who require nursing care may become aggressive and cause problems for the care providers or other patients, in addition to themselves. The direct cause as to why people who require nursing care act aggressively is likely to be discomfort and anxiety about being touched inside the oral cavity.
  2. Measures: sufficient explanation of treatment
    It is necessary to fully inform the patient that one is a dentist who is going to perform dental treatment, and to continue to inform the patient repeatedly during treatment that the treatment is being provided. On the other hand, these measures may not be sufficient for patients who are highly irritable. As these patients are often prescribed antianxiety agents or antipsychotic drugs, treatment should be provided with coordination while the effects of the drugs persist.
10. **Others**
  1. Accidental ingestion and aspiration
    It may be difficult in some patients who are receiving nursing care to check for loosening or falling of the superstructure due to poor cleaning inside the oral cavity (stagnation of food residue), deterioration in swallowing reflex and so forth. If accidental ingestion or aspiration is suspected as the superstructure comes off inside the oral cavity, dental X-ray photographs of the oral cavity and X-ray photographs of the chest should be taken for confirmation.
  2. Problems in changing to removable denture
    One useful way to improve the ease of cleaning is to remove the implant superstructure and change it to a removable denture or implant overdenture [18].
  3. Failure to determine the implant manufacturer
    If the type of implant body is unknown, troubles may occur frequently as proper measures cannot be taken during visiting dental care. Every effort should be made to identify the type.

**Table 1:** Maintenance issues of elderly patients with implant treatments in dental visiting

	Complications		Measures
Biological complications	• Inflammations around implants		• Reinforcement of brushing
			• Mechanical cleaning (CIST: A)
			• Sterilization treatment (CIST: B)
			• Antibacterial agent treatment (CIST: C)
			• Surgical treatment
	• Poor cleaning		1. Instructions on cleaning for nursing care providers
			2. Professional care and instructions on cleaning for nursing care providers
	• Wounds		1. Removal of the superstructure
			2. Shape of the sharp edges
	• Bite wounds on others		1. Sufficient explanation of treatment
			2. Treatment under the effect of antianxiety agents or antipsychotic drugs
	• Accidental ingestion and aspiration		• Dental X-ray photographs of the oral cavity and X-ray photographs of the chest
Prosthetic complications	• Implant fracture		
	Fracture in neck part of implant		1. Reattachment of the upper structure or attachment of a healing abutment
			2. Sealing
	Fracture in stem part of implant		• Remove (follow-up of osseointegrated implants)
	• Screw loosening or fracture		
	Screw loosening		1. Re-fastened
			2. Removal of the superstructure → sealing
			3. Follow-up
	Screw fracture		1. Remove
			2. Sealing
	• Fracture in facing part		
	Anterior teeth		• Conventional repair
	Molar teeth		• Shape of the sharp edges
	• Loss of temporary sealing on access hole		• Selection of hard type temporary sealing material
	• Lack of retention of the prosthesis		1. Occlusal adjustment
			2. Removal of the superstructure → sealing
			3. Cementation

## Limitations

The measures for implant troubles during visiting dental care described in this paper are mostly the opinions of the 12-member expert panel based on their clinical experience, and are not based on solid evidence.

We hope this paper will be validated in the future by properly designed clinical studies and further accumulation of evidence, and that medical care guidelines on implant management including oral care for the elderly and visiting dental care will eventually be established.

## Conclusion

Considering the fact-finding survey on implant treatments in visiting dental care and the difficulties in handling implant troubles at the site of medical care, this paper makes the following suggestions:

1. Not only medical professionals, but also nursing care providers and patients should fully understand implant treatments for the elderly and that the condition of the oral cavity of patients (25–29), the systemic conditions, economic environment and so forth (30) change with time.
2. To discover implant troubles and take measures at an early stage, it is essential that continuous maintenance is provided by the doctor who performed the implant or by medical professionals with sufficient knowledge and experience to be able to handle implant treatments.
3. In the future, not only oral implant specialists, specialized dental hygienists, and specialized dental technicians, but also many people working in dentistry will need to deepen their understanding of nursing care and improve their knowledge on oral implants in the elderly and their ability to manage implants.

## Data Availability

All data presented in manuscript are available for publication. This article is based on a position paper first reported in the *Journal of Japanese Society of Oral Implantology* 2018;31(4):3–21.
